# The Curious History of L'Hotel-Dieu De Paris


**Published:** 1913-03-08

**Authors:** 


### THE CURIOUS HISTORY OF L'HOTEL-DIEU DE PARIS.*

### I.—ITS FOUNDATION AND EARLY DAYS.

The credit of founding the Hôtel-Dieu de Paris is commonly given to Bishop Inchad, who flourished in the ninth century, but tradition has it that the institution is of still more ancient date, and that it owed its inception to St. Landry, who was Bishop of Paris two centuries earlier. Constructed at the steps of Notre-Dame, *ante portam ecclesiæ*, the Hôtel-Dieu, before taking in the twelfth century the name *Maison-Dieu de Paris—Domus Dei Parisiensis* (whence it derived its present appellation), was called successively *Hospice Saint-Christophe* and *Hospice Notre-Dame*. Demolished in 1184 because of the necessity of improving the city's sewerage works, the original fabric was replaced by new buildings constructed along the lesser arm of the Seine, from the Pont au Double to the Petit-Pont. A whole series of kings and queens were interested in this rebuilding. Philippe-Auguste erected the first salle or ward of the new hospital, the Salle Saint-Denis,† and among other benefits bestowed upon it "all the straw of his room and his house in Paris at such times as he went to lie elsewhere." A little later Blanche de Castille added the Salle Saint-Thomas,† and her son Saint-Louis,† the Salle Neuve or Jaune, which flanked the river bank and terminated near the Petit-Pont in two chapels, dedicated to St. Agnes. This King also took the institution under his patronage, and gave it revenues and important privileges. Louis XI. completed the work of his predecessors, and adorned the chapels with two fine doors. Certain wealthy private individuals also became benefactors. Oudart de Mocreux, a money-changer and burgess of Paris, in 1280 restored and enlarged the entrance to the hospital on the Place du Parvis, as well as the chapel. Cardinal Duprat, Papal Legate, built in 1535 the Salle Sainte-Marthe,† or du Légat, near and parallel to the Salle Saint-Louis. "It will be a very large one," said Francis I., "if it is to contain all those he has made wretched." It was, in fact, a beautiful building, which could accommodate a hundred beds with ease. Thus in the sixteenth century the hospital was of some importance and of real architectural beauty. It drew the attention to its two extremities, the one near the Petit-Pont, the other, its entrance, on the Place du Parvis. The Salle Neuve and the Salle du Légat stretched along the river's bank between these extremities to raise their gable-ends in juxtaposition on the Rue du Marché Palu. That of the former consisted of two doors placed side by side with pointed archways, the "beaux portaulx" of Louis XI., peopled with statuettes, surmounted by gables and slender pinnacles, and crossed by a balustrade. The doors gave access to the chapels of St. Agnes. Together these buildings formed two magnificent examples of the styles of the Middle Ages and of the Renaissance.

The entrance to the hospital, close to Notre-Dame, was near to where the statue of Charlemagne now stands, though a little farther away from the river. It consisted of a small building with a pointed roof, surmounted by a bell-turret. Two pointed doors gave access to it, of which the principal was supplied with an overhanging porch of timber and forged iron surmounted by a *mâchicoulis* or balcony with holes for firing through. The same entrance admitted to the chapel, which, pierced by large pointed windows delicately chiselled, was a veritable architectural gem.

In front of and facing the entrance of the Hôtel-Dieu stood a statue of stone representing a tall sad-looking individual holding in one hand a book and in the other a stick around which serpents were entwined. Its origin is lost in obscurity. It had stood in the Place du Parvis from times immemorial, and no one knew whom it was meant to represent. To this enigma in stone the people of Paris had given the name *Grand Jeusneur* or *Maître Pierre le Jeusneur*, and it played a part in the life of the city. On almost all occasions, and especially in moments of popular excitement, it was this person who was supposed to express his opinions in irresponsible fashion by means of broad-sheets, lampoons, etc., secretly distributed broadcast. His career as a public character came to an end in 1748, when the Place du Parvis was enlarged. The internal arrangement of the hospital was simple. Below, in a collection of cellars were—bitter irony!—the *chambres aisées* for lying-in women. The *salles* were covered in by vaulted roofs. The smaller ones consisted of a single nave, and resembled Gothic chapels. The larger, with their many aisles, vast interiors, and lofty supporting pillars, were more like cathedrals. Below the *salles*, and serving as foundations for them, were the famous *cagnards*, dismal retreats badly illuminated from the river by a series of openings, and occupied by the *salle des Morts*, the large and small washhouses, and the bleaching rooms. They also formed the *port de l'Ostel-Dieu*, at which were drawn up the boats conveying provisions to the hospital, and from which embarked the monks when " going to visit the estate or to collect rents." The seventeenth century saw the climax of the development and grandeur of the Hôtel-Dieu. The old buildings had been restored or reconstructed by the architect Vellefeux, and new structures added. The Pont au Double was built in 1626.

A part of the bridge was set apart for the use of foot-passengers, from whom a *double tournois* was exacted as toll, whence the name " Pont au Double." Having annexed the bed of the river in this way, the hospital commandeered the left bank also, and in 1651 the Salle Saint-Charles† was built. This started at the Pont au Double, descended towards the Petit-Pont, and was enlarged to reach this last in 1714. To facilitate communication between the new building and the Salle Saint-Thomas, as well as to provide a promenade for the inmates, a new bridge, the Pont Saint-Charles, was then thrown over the river. The sight presented by this arm of the river at that time must have been most picturesque, closed in as it was on both sides by the high buildings, crossed at one end by the Pont au Double, on which rested the Salle du Rosaire, at the other by the Petit-Pont crowded with houses, and divided in the middle by the Pont Saint-Charles. Where nowadays stretch green lawns and blooming gardens at that time could be seen a river, a dock, and the *Port de l'Ostel-Dieu*.

The eighteenth century brought this change. Three fires, terrible and all-devouring, followed each other in quick succession, and all but razed the hospital to the ground. Of what was left, the Pont au Double was demolished in the reign of Charles X., the Salle Saint-Charles was reduced in size, and the bridge of that name destroyed in 1836. The few ruins of the beautiful front of the Chapels of St. Agnes and the Salle du Légat were allowed to remain for a few years, but were finally removed by the architect Clavareau. After the last fire in 1772 the committee appointed by Louis XV. to inquire into the rebuilding of the hospital decided to transfer it to a new site, but the community of the hospital objected, and it was reconstructed where it had stood for so long. It consisted of an entrance hall and three parallel buildings connected by two covered-in bridges. The first building occupied the right bank of the river, and was erected on the *cagnards*. The second was situated between the lesser arm and the rue de la Bûcherie, and the third on the opposite bank of the river. The only interesting portion of the hospital from the architectural standpoint was the entrance hall. Built in 1803 by Clavareau, it was a square and low pavilion, of which the severe portico was composed of Doric columns supporting a frieze and pediment without ornaments. It was approached by a flight of steps.

This little Greek temple must have contrasted strangely with the great Gothic cathedral situated so near it. The idea of moving the hospital to a new site had, however, never been quite lost sight of, and the architect Gilbert presented no less than three plans to that end between 1853 and 1861. But it fell to another plan, that of Diet, to be accepted by Baron Haussmann, then Prefect of the Seine. Work was begun at once under the patronage of Napoleon III., but was interrupted by the war of 1870, and it was not until 1877 that the hospital was ready to be opened by Marshal de MacMahon. Its cost had amounted to 36,400,000 francs (£1,456,000). Though it had found new quarters, it still remained faithful to the city in which its history had been made. The work of demolishing the old building began as soon as the new one was completed, and only a little time since the last traces of an historic monument were swept away.

* This account of the history of the Hôtel-Dieu and its connection with medical teaching in France has been adapted from an inaugural lecture delivered by Professor Gilbert, Professor of Clinical Medicine at the Hôtel-Dieu, on November 19, 1910, and published in *Paris Médical*, No. 1.

† The names marked with a dagger have been given to wards in the present Hôtel-Dieu.

